# Homozygous PNPLA6 Mutation (p.Arg1183Trp) Associated With Isolated Cerebellar Ataxia: A Familial Case Report

**DOI:** 10.7759/cureus.107039

**Published:** 2026-04-14

**Authors:** Ahmad Alawadhi, Ali Almarzooqi, Maria Khan, Suhail Alrukn

**Affiliations:** 1 Neurology, Rashid Hospital, Dubai, ARE; 2 Neurology, Mohammed bin Rashid University of Medical and Health Sciences, Dubai, ARE

**Keywords:** cerebellar ataxia, consanguinity, genetic mutation, hereditary spastic paraplegia, neurodegeneration, pnpla6

## Abstract

This case report describes a novel presentation of homozygous PNPLA6 gene mutation (c.3547C>T; p.Arg1183Trp) manifesting as isolated cerebellar ataxia in two male first cousins from an Emirati family, characterized by cerebellar ataxia without systemic manifestation. This report highlights the implications for genetic diagnosis and counseling in families with consanguinity.

Two affected male first cousins (ages 53 and 61 years) were retrospectively identified through our tertiary-care neurology clinic's medical registry. Both patients underwent standardized neurological examination, brain magnetic resonance imaging (MRI), and next-generation sequencing of ataxia-related genes. The family history was assessed in consenting relatives.

Both patients presented with insidious onset dysarthria, progressive truncal and limb ataxia, wide-based gait, dysmetria, and bilateral dysdiadochokinesia. Brain MRI (both T1-weighted and T2-weighted sequences) demonstrated isolated cerebellar vermian and hemispheric atrophy with increased cerebrospinal fluid (CSF) spaces. No endocrine, retinal, or systemic abnormalities were identified. Genetic testing confirmed the same homozygous PNPLA6 variant (c.3547C>T; p.Arg1183Trp). The patients were offered supportive physiotherapy to improve balance and fall precautions, as well as genetic counseling for family planning.

This familial case report expands the recognized phenotypic spectrum of PNPLA6-related disorders by demonstrating predominantly cerebellar ataxia without endocrine or retinal involvement. These findings underscore the importance of genetic screening in unexplained adult-onset ataxia, particularly in consanguineous families, even in the absence of classic syndromic features.

## Introduction

Cerebellar ataxia is a heterogeneous group of disorders characterized primarily by motor coordination difficulties due to cerebellar dysfunction [1]. This group can be divided into genetic, sporadic, and acquired forms. Early-onset ataxias often present before the age of 25 years and commonly follow an autosomal recessive inheritance pattern [2]. Symptoms typically include gait instability, limb incoordination, dysarthria, and oculomotor abnormalities. Many cerebellar ataxias involve extracerebellar manifestations, such as cranial nerve abnormalities and peripheral neuropathy, which pose diagnostic and treatment challenges [2,3].

Patatin-like phospholipase domain-containing protein 6 (PNPLA6), originally called neuropathy target esterase (NTE), was initially associated with spastic paraplegia type 39 (SPG39) and later with a broad spectrum of neurodegenerative disorders such as Gordon-Holmes syndrome (GHS), Boucher-Neuhauser syndrome (BNS), Oliver-McFarlane syndrome (OMS), and Laurence-Moon syndrome (LMS). The continuum of clinical features of these syndromes varies from cerebellar ataxia, hypogonadotropic hypogonadism, chorioretinal dystrophy, spastic paraplegia, muscle wasting, peripheral neuropathy, to cognitive impairment [4,5].

PNPLA6 was initially identified to play a key role in organophosphate-induced delayed neuropathy, which is a degenerative condition associated with organophosphate exposure [6]. The gene follows an autosomal recessive pattern of inheritance and encodes the membrane protein NTE.

Nanetti et al. conducted a study in which genetic screening was performed in 292 patients presenting with ataxia or spastic paraplegia using a probe-based customized gene panel covering over 200 genes associated with spinocerebellar disorders. Out of the 292 patients screened, 6 novel and 4 recurrent PNPLA6 gene variants were identified. Six patients had juvenile-onset (<18 years of age), and two had adult-onset disease. The most common finding was cerebellar atrophy on brain MRI, which was more pronounced in the superior cerebellar vermis lobules, followed by hypogonadotropic hypogonadism [7].

## Case presentation

Case 1

History

A 53-year-old, right-handed man presented with progressive imbalance over the preceding year. He reported frequent falls and difficulty handling utensils due to hand tremor and incoordination. He denied dizziness, motor or sensory weakness, numbness, tingling sensations, alcohol use, or other substance abuse. His past medical history was notable for degenerative disc disease at C4-C6 without spinal cord compression.

Neurological Examination

The patient exhibited dysarthric speech. Cranial nerve examination revealed horizontal gaze-evoked nystagmus bilaterally and non-fatigable horizontal nystagmus. Visual acuity and visual fields were normal. Other cranial nerves were intact. Motor examination demonstrated normal tone, full power (5/5) in all muscle groups, and normal deep tendon reflexes. Sensory examination was normal to all modalities. Coordination testing revealed left-sided dysmetria on finger-to-nose testing, dysdiadochokinesia, and impaired heel-to-shin testing. Gait examination demonstrated a wide-based ataxic gait with an inability to perform tandem gait.

Investigations

Laboratory investigations, including thyroid function tests, vitamin E levels, liver and kidney function tests, copper and ceruloplasmin levels, heavy metal screening, and autoimmune panels, were all normal. Nerve conduction studies and electromyography (EMG) were performed to evaluate for associated peripheral neuropathy, and were normal.

Genetic testing for common spinocerebellar ataxias and fragile X-associated tremor/ataxia syndrome (FXTAS) was negative. Comprehensive next-generation sequencing using an ataxia gene panel (932 genes) revealed a homozygous variant c.3547C>T; p.Arg1183Trp in the PNPLA6 gene.

Endocrine evaluation, including follicle-stimulating hormone (FSH), luteinizing hormone (LH), testosterone, and free testosterone levels, was within normal ranges, ruling out hypogonadotropic hypogonadism. The patient had fathered five children, confirming prior fertility. Ophthalmological examination was normal with no evidence of chorioretinal degeneration.

Imaging

**Figure 1:**
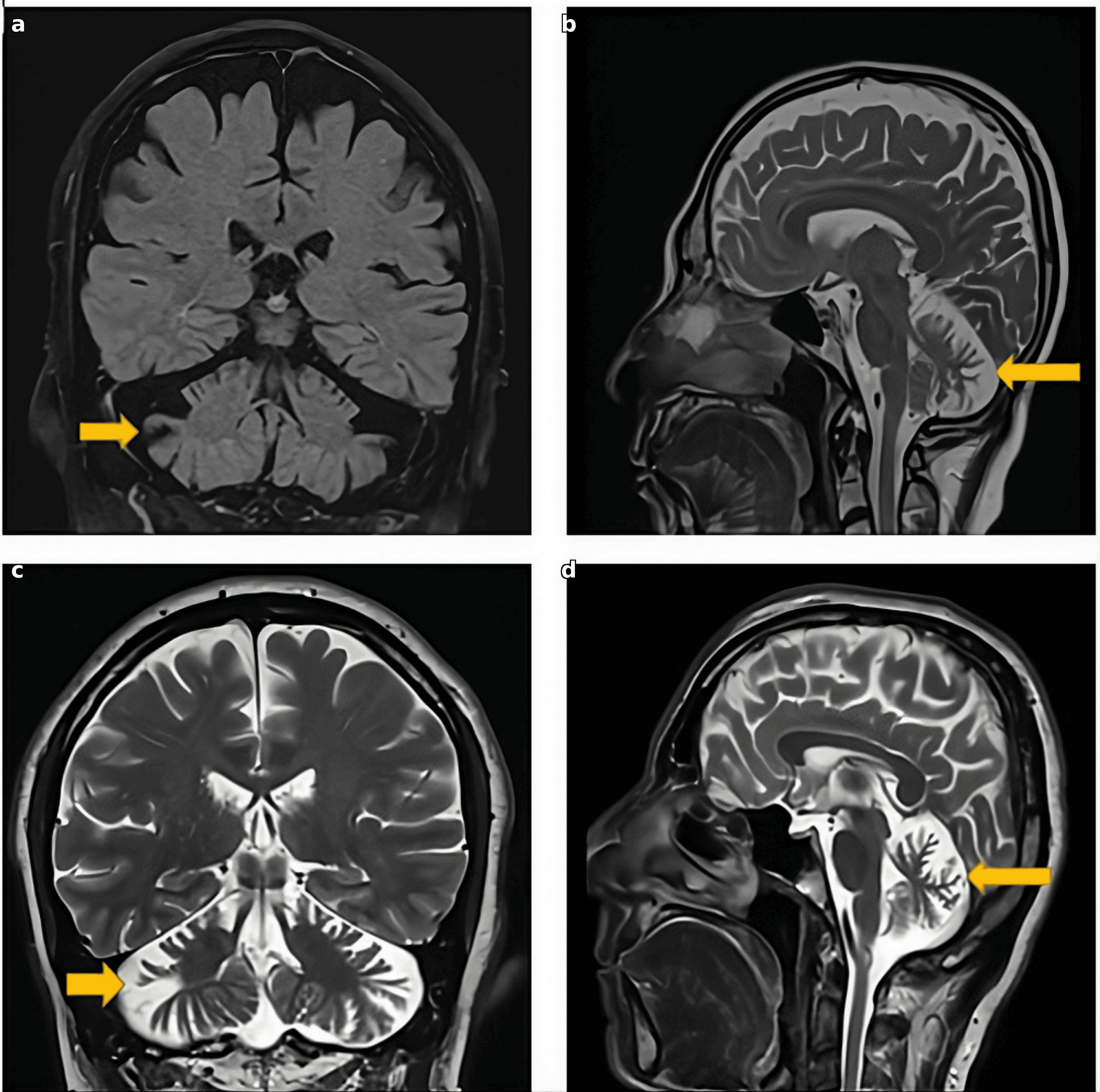
Brain MRI of Case 1 demonstrating cerebellar atrophy (a) Coronal T1-weighted image showing cerebellar vermian and hemispheric atrophy with prominence of cerebellar folia and widening of cerebellar sulci (arrow indicates cerebellar atrophy). (b) Sagittal T1-weighted image demonstrating cerebellar atrophy with enlargement of the fourth ventricle and increased cerebrospinal fluid spaces (arrow). (c) Coronal T2-weighted image showing cerebellar atrophy with prominent cerebellar sulci (arrow). (d) Sagittal T1-weighted image demonstrating cerebellar vermian atrophy with fourth ventricular enlargement (arrow).

Brain MRI demonstrated cerebellar atrophy characterized by prominence of cerebellar folia, widening of cerebellar sulci, and enlargement of the fourth ventricle. The atrophy was most pronounced in the cerebellar vermis and hemispheres bilaterally. T1-weighted coronal images showed cerebellar volume loss with increased cerebrospinal fluid (CSF) spaces (Figure 1). T1-weighted sagittal images demonstrated vermian atrophy with fourth ventricular enlargement (Figure 1). No signal abnormalities were noted in the brainstem, basal ganglia, or cerebral hemispheres.

Management and Follow-Up

The patient was referred for physiotherapy focusing on balance training and fall-prevention strategies. Genetic counseling was provided to discuss inheritance patterns, recurrence risks, and family screening recommendations.

Case 2

History

A 61-year-old, right-handed man (first cousin of Case 1) presented with progressive gait instability over the preceding 18 months. He reported frequent falls and urinary incontinence. He denied alcohol use, cognitive impairment, spatial disorientation, pain, motor or sensory weakness, or numbness. He had no significant past medical history. Four months after initial presentation, he developed progressive neurological deterioration with new-onset fecal incontinence and dysphagia.

Neurological Examination

Cognitive functions were intact. The patient exhibited dysarthric speech. Cranial nerve examination revealed horizontal gaze-evoked nystagmus bilaterally and restricted extraocular movements. Visual acuity and visual fields were normal. Other cranial nerves were intact. Motor examination demonstrated mildly increased tone in all limbs, full power (5/5) in all muscle groups, and normal deep tendon reflexes. Sensory examination was normal to all modalities. Coordination testing revealed bilateral dysmetria on finger-to-nose testing, dysdiadochokinesia, and impaired heel-to-shin testing bilaterally. Gait examination demonstrated a wide-based ataxic gait.

Investigations

Laboratory investigations, including thyroid function tests, liver function tests, kidney function tests, and autoimmune screening, were normal. Nerve conduction studies and EMG were normal, excluding peripheral neuropathy.

Following the genetic diagnosis in his first cousin (Case 1), targeted genetic testing was performed, confirming the same homozygous PNPLA6 variant (c.3547C>T; p.Arg1183Trp).

Endocrine evaluation was normal, and the patient had fathered children prior to disease onset, confirming prior fertility. Ophthalmological examination showed no evidence of chorioretinal degeneration.

Imaging

**Figure 2:**
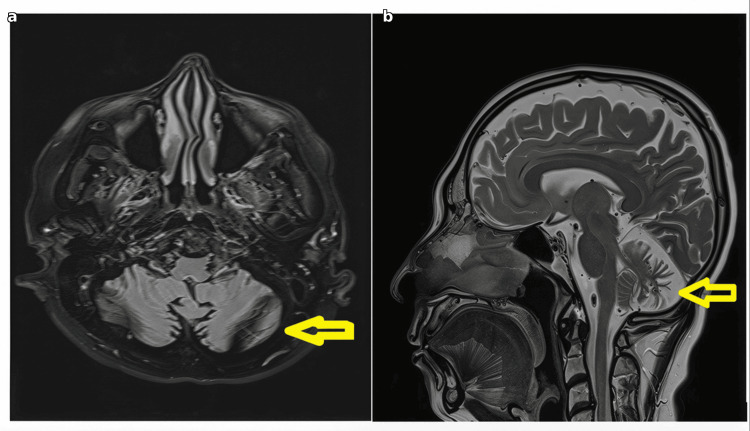
Brain MRI of Case 2 demonstrating cerebellar atrophy (a) Axial T2-weighted image showing cerebellar atrophy with prominence of cerebellar sulci (arrow indicates cerebellar atrophy). (b) Sagittal T1-weighted image demonstrating cerebellar vermian atrophy with enlargement of the fourth ventricle (arrow).

Brain MRI demonstrated cerebellar atrophy characterized by prominence of cerebellar folia, widening of cerebellar sulci, and enlargement of the fourth ventricle. The pattern was similar to Case 1, with predominant involvement of the cerebellar vermis and hemispheres. T2-weighted axial images showed cerebellar atrophy with prominent sulci (Figure 2). T1-weighted sagittal images demonstrated vermian atrophy with fourth ventricular enlargement (Figure 2). No signal abnormalities were noted in the brainstem, basal ganglia, or cerebral hemispheres.

Management and Follow-Up

**Figure 3:**
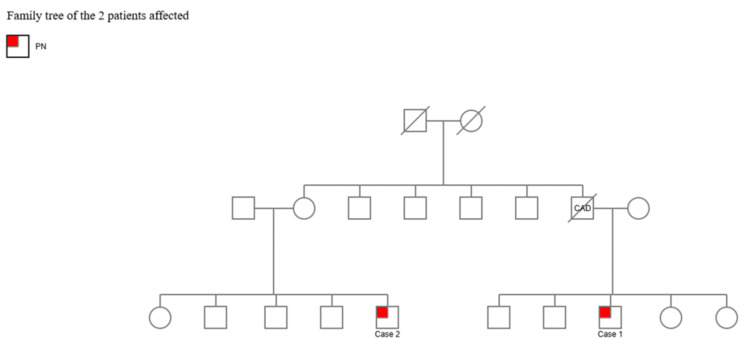
Pedigree of the Emirati family demonstrating autosomal recessive inheritance Squares represent males, circles represent females, while filled symbols represent affected individuals. The two affected first cousins share the same homozygous PNPLA6 mutation (c.3547C>T; p.Arg1183Trp). PN: patient with PNPLA6 gene mutation; CAD: coronary artery disease (cause of death)

The patient was referred for physiotherapy and occupational therapy. Fall-prevention strategies and home safety modifications were recommended. Genetic counseling was provided. Additional family screening was recommended for at-risk relatives (Figure 3).

Comparative analysis of both cases

Both patients are male first cousins from a consanguineous Emirati family sharing the same homozygous PNPLA6 mutation. Key differences include age of onset (53 vs. 61 years), with Case 1 presenting at a younger age. Case 2 demonstrated more rapid clinical progression, developing autonomic dysfunction (urinary and fecal incontinence) and bulbar involvement (dysphagia) within 18 months of symptom onset, whereas Case 1 showed a more slowly progressive course limited to cerebellar features after one year.

Neurological findings were similar in both cases, including dysarthria, horizontal gaze-evoked nystagmus, dysmetria, dysdiadochokinesia, and ataxic gait. Case 2 additionally demonstrated restricted extraocular movements and bilateral (rather than unilateral) coordination deficits, suggesting more extensive cerebellar involvement. Case 2 also exhibited mildly increased tone, which was absent in Case 1.

MRI findings were comparable in both cases, showing isolated cerebellar vermian and hemispheric atrophy with no supratentorial involvement. Both patients had normal endocrine function, prior fertility, and normal ophthalmological examinations, confirming the absence of hypogonadotropic hypogonadism and chorioretinal degeneration. Table 1 provides a detailed comparison of clinical features between the two cases.

**Table 1:** Clinical comparison of both cases This table compares the clinical features, neurological findings, imaging results, and genetic characteristics between Case 1 (53-year-old male) and Case 2 (61-year-old male), both first cousins sharing the same homozygous PNPLA6 mutation. EOM: extraocular movements; MRI: magnetic resonance imaging

Feature	Case 1 (53 years)	Case 2 (61 years)
Symptom Duration	1 year	18 months
Presenting Symptoms	Imbalance, falls, hand incoordination	Imbalance, falls, urinary incontinence
Disease Progression	Slowly progressive, pure cerebellar	Rapid progression, cerebellar + autonomic + bulbar
Dysarthria	Present	Present
Nystagmus	Horizontal gaze-evoked, bilateral	Horizontal gaze-evoked, bilateral + restricted EOM
Limb Ataxia	Left-sided dysmetria, dysdiadochokinesia	Bilateral dysmetria, dysdiadochokinesia
Muscle Tone	Normal	Mildly increased in all limbs
Autonomic Dysfunction	Absent	Present (urinary and fecal incontinence)
Bulbar Involvement	Absent	Present (dysphagia)
MRI Findings	Isolated cerebellar vermian and hemispheric atrophy	Isolated cerebellar vermian and hemispheric atrophy
Fertility Status	Father of 5 children; hormones normal	Fathered children; hormones normal
Ophthalmology	Normal; no chorioretinal degeneration	Normal; no chorioretinal degeneration
PNPLA6 Genotype	Homozygous c.3547C>T; p.Arg1183Trp	Homozygous c.3547C>T; p.Arg1183Trp

## Discussion

Autosomal recessive cerebellar ataxias (ARCA) are a diverse group of genetic disorders marked by progressive cerebellar dysfunction. While most PNPLA6-related disorders present with multisystem involvement, including hypogonadotropic hypogonadism, chorioretinal degeneration, and spastic paraplegia, our cases represent a unique presentation with isolated cerebellar dysfunction [8]. Tarnuzzer et al. reported PNPLA6 gene mutations in four out of six patient families with BNS, confirming the genetic basis of this disorder [8]. Beyond BNS, PNPLA6 mutations have been implicated in other syndromic presentations, including OMS, GHS, and various forms of hereditary spastic paraplegia (HSP) [9,10].

Teive et al. reported a 78-year-old male patient presenting with insidious-onset cerebellar ataxia and hypogonadism. Genetic analysis revealed a homozygous PNPLA6 mutation, categorizing the patient as having GHS [11]. In contrast, our patients exhibited isolated cerebellar features without evidence of hypogonadism, retinal degeneration, or spastic paraplegia. Both patients had evidence of prior fertility (Case 1 had five children; Case 2 had fathered children before disease onset), and hormonal testing demonstrated normal gonadotropin and testosterone levels, arguing against hypogonadotropic hypogonadism. Ophthalmological screening was unremarkable in both cases, with no evidence of chorioretinal degeneration.

Brain MRI in both patients revealed isolated cerebellar vermian and hemispheric atrophy without supratentorial involvement. The cerebellar atrophy was characterized by prominence of cerebellar folia, widening of cerebellar sulci, and enlargement of the fourth ventricle, consistent with cerebellar degeneration. No signal abnormalities were noted in the brainstem, basal ganglia, or cerebral hemispheres. Genetic testing confirmed the same homozygous PNPLA6 variant (c.3547C>T; p.Arg1183Trp) in both patients, supporting the diagnosis of PNPLA6-related cerebellar ataxia. Genetic counseling was offered to the patients and their families to discuss inheritance patterns, recurrence risks, and implications for family planning.

This case report presents a clinically relevant and potentially novel phenotype of PNPLA6 mutation manifesting as isolated cerebellar ataxia. The presentation differs from the classically described syndromes associated with PNPLA6 mutations, which typically include multisystem involvement. The absence of hypogonadism, chorioretinal degeneration, and spastic paraplegia in our patients suggests phenotypic variability within PNPLA6-related disorders, which may be influenced by modifier genes, environmental factors, or the specific mutation involved. Table 2 lists the clinical spectrum of PNPLA6 mutation-associated disorders.

**Table 2:** Clinical spectrum of PNPLA6 mutation-associated disorders This table summarizes the key clinical features and additional findings of various PNPLA6-related syndromes reported in the literature, demonstrating the broad phenotypic spectrum associated with PNPLA6 mutations.

Disorder	Key Clinical Features	Additional Findings	References
Boucher-Neuhauser Syndrome	Cerebellar ataxia, hypogonadotropic hypogonadism, chorioretinal dystrophy	Cognitive impairment, scoliosis	Synofzik et al. [4]
Gordon-Holmes Syndrome	Cerebellar ataxia, hypogonadotropic hypogonadism	Spasticity, peripheral neuropathy, intellectual disability	Synofzik et al. [4]
Hereditary Spastic Paraplegia (SPG39)	Spasticity, cerebellar ataxia	Peripheral neuropathy, intellectual disability	Elsayed et al. [12]
Oliver-McFarlane Syndrome	Cerebellar ataxia, chorioretinal dystrophy, hypogonadism	Scalp alopecia or trichomegaly, growth hormone deficiency	Hufnagel et al. [10]
Laurence-Moon Syndrome	Retinal dystrophy, hypogonadism	Intellectual disability, spastic paraplegia	Hufnagel et al. [10]

## Conclusions

PNPLA6 mutations have been implicated in various neurodegenerative disorders with overlapping phenotypes, including BNS, GHS, OMS, and HSP. The clinical spectrum ranges from isolated cerebellar ataxia to complex multisystem disorders involving hypogonadism, retinal degeneration, and cognitive decline. Our cases represent a unique presentation of PNPLA6 mutation with isolated cerebellar ataxia without systemic features, expanding the known phenotypic spectrum.

The diagnosis of PNPLA6-related disorders relies on genetic testing, particularly in cases of unexplained adult-onset ataxia with or without additional features. Early genetic identification enables appropriate genetic counseling and facilitates informed family planning decisions. Longitudinal follow-up studies are needed to determine whether additional features develop over time in patients presenting initially with isolated cerebellar ataxia. Clinicians should maintain a high index of suspicion for PNPLA6 mutations in patients presenting with progressive cerebellar ataxia, especially in familial clusters or consanguineous families. Comprehensive genetic screening should be considered even in the absence of classic syndromic features, as phenotypic expression can be highly variable. Further research is needed to better understand genotype-phenotype correlations and to develop targeted therapeutic strategies for PNPLA6-related disorders.
